# Heat treatment prior to testing allows detection of antigen of *Dirofilaria immitis* in feline serum

**DOI:** 10.1186/1756-3305-7-1

**Published:** 2014-01-13

**Authors:** Susan E Little, Melissa R Raymond, Jennifer E Thomas, Jeff Gruntmeir, Joe A Hostetler, James H Meinkoth, Byron L Blagburn

**Affiliations:** 1 Department of Veterinary Pathobiology, Center for Veterinary Health Sciences, Oklahoma State University, Stillwater, OK 74078, USA; 2 Bayer HealthCare, Animal Health, Shawnee Mission, Shawnee, KS, USA; 3 Department of Veterinary Pathobiology, College of Veterinary Medicine, Auburn University, Auburn, AL, USA

**Keywords:** Antigen test, Cat, *Dirofilaria immitis*, Heartworm, Heat treatment

## Abstract

**Background:**

Diagnosis of *Dirofilaria immitis* infection in cats is complicated by the difficulty associated with reliable detection of antigen in feline blood and serum samples.

**Methods:**

To determine if antigen-antibody complex formation may interfere with detection of antigen in feline samples, we evaluated the performance of four different commercially available heartworm tests using serum samples from six cats experimentally infected with *D. immitis* and confirmed to harbor a low number of adult worms (mean = 2.0). Sera collected 168 (n = 6), 196 (n = 6), and 224 (n = 6) days post infection were tested both directly and following heat treatment.

**Results:**

Antigen was detected in serum samples from 0 or 1 of 6 infected cats using the assays according to manufacturer’s directions, but after heat treatment of serum samples, as many as 5 of 6 cats had detectable antigen 6–8 months post infection. Antibodies to *D. immitis* were detected in all six infected cats by commercial in-clinic assay and at a reference laboratory.

**Conclusions:**

These results indicate that heat treatment of samples prior to testing can improve the sensitivity of antigen assays in feline patients, supporting more accurate diagnosis of this infection in cats. Surveys conducted by antigen testing without prior heat treatment of samples likely underestimate the true prevalence of infection in cats.

## Background

Detection of antigen of *Dirofilaria immitis* is a sensitive and specific means of diagnosing heartworm infection in dogs, but these tests have been considered much less reliable in cats [1-3]. Lack of antigen detection in cats has been attributed to low circulating antigenemia due to the low number of worms often seen in feline infections, a higher likelihood of male only infections, and stunted development of *D. immitis* adults in an aberrant host [4-6]. In dogs, these assays are considered capable of detecting infections with as few as 1 – 3 adult female *D. immitis*[1,7].

When antigen-based assays were first developed for detecting *D. immitis* in dogs, serum was pre-treated with heat and/or EDTA to destroy immune complexes prior to testing, as antigen-antibody complexes were recognized as inhibiting detection of antigen in some canine samples [8-10]. However, this step is no longer included in protocols of commercial *D. immitis* antigen tests, including those labeled for use in cats. We recently described that heat treatment of canine serum samples prior to testing can reveal antigen of *D. immitis*, a phenomenon which may interfere with detection of infection in as many as 7% of dogs in the southern United States [S. Little, unpublished data]. In canine serum samples which test false negative on commercial assays, antigen is apparently trapped in immune complexes, preventing detection. Once these complexes are disrupted, the assays are able to accurately detect antigen [8-10].

To determine the role antigen blocking may play in detection of *D. immitis* antigen in feline samples, we tested serum samples from cats experimentally infected with a low number of heartworms with four different commercially available assays before and after heat treatment of sera.

## Methods

### Samples

Large volume serum samples were available from six (3 male and 3 female) domestic short-haired cats experimentally infected at 10 months of age with *Dirofilaria immitis* by subcutaneous inoculation of third-stage larvae (L_3_) and confirmed to be infected by recovery of adult worms at necropsy or confirmation of histological lesions. Briefly, trickle infection of a total of 100 L_3_ of *D. immitis* was performed by subcutaneous inguinal inoculation of each cat a total of four times, on study days 7, 14, 21, and 28, with 25 L_3_ (Missouri strain) harvested shortly prior to inoculation from infected *Aedes aegypti* mosquitoes (Liverpool strain). Whole blood samples were collected from the jugular vein or, rarely, the cephalic vein, of each cat on days 84, 112, 140, 168, 196, and 224 directly into vacuum tubes containing either EDTA or no additive. Cats were cared for through Oklahoma State University’s Association for Assessment and Accreditation of Laboratory Animal Care-accredited animal resources program throughout the study; all research procedures followed a detailed animal care and use protocol approved by Oklahoma State University’s Institutional Animal Care and Use Committee. Anti-coagulated whole blood was assayed for microfilaria by modified Knott’s test and by real-time PCR for *Wolbachia* spp. as previously described [11,12]. For tubes without additive, blood was allowed to clot, the serum separated by centrifugation, placed into aliquots, and stored at −80°C until further use.

### Antibody testing

Antibody testing was conducted using a commercial assay (Solo Step® FH, HESKA) according to manufacturer’s instructions, and by a fee-for-service reference laboratory (ANTECH Diagnostics) using a commercially available microtiter plate assay (Synbiotics Corporation, Zoetis). The reference laboratory tested each sample in triplicate and provided optical density (O.D.) results from each well as determined on spectrophotometry as well as corresponding positive and negative control sample wells.

### Antigen testing

All antigen testing was conducted using commercial assays according to manufacturers’ instructions, with the exception that samples were tested before and after heat treatment on each assay. For heat treatment, serum samples were placed in a heat block at 103°C for 10 minutes, the resultant coagulum centrifuged, and the supernatant used in each commercial assay. Test kits evaluated before and after heat treatment included enzyme linked immunosorbent assay (ELISA) in microtiter plate formats (DiroCHEK®, Synbiotics Corporation, Zoetis; PetChek® Heartworm PF Antigen Test, IDEXX Laboratories, Inc.), membrane bound ELISAs (SNAP® Feline Heartworm® Test, IDEXX Laboratories, Inc.), and lateral flow immunochromatographic tests (WITNESS® HW, Synbiotics Corporation, Zoetis). In addition, O.D. readings were obtained by spectrophotometry before and after heat treatment for one of the microtiter plate assays (PetChek® Heartworm PF Antigen Test, IDEXX Laboratories, Inc.) according to manufacturer’s directions.

### Comparison of test results

Optical density from microtiter plate assay (PetChek® Heartworm PF Antigen Test, IDEXX Laboratories, Inc.) and mean O.D. of triplicate antibody tests (ANTECH Diagnostics) for each cat for all days on which antigen was detected in any one cat and for cats on each of three individual study days (study days 168, 196, and 224) were compared using one-way ANOVA in Excel 2007 (Microsoft Office) with significance assigned at alpha = 0.05 [13]. An antigen/antibody ratio was also determined for each cat on days 168, 196, and 224 using O. D. and mean O. D., respectively.

## Results

All six cats developed infection with *D. immitis* as evidenced by detection of classic lesions on histopathologic examination or recovery of adult worms at necropsy. A total of 1 – 6 adult *D. immitis* were collected from 5 cats; adult worms were not recovered from the remaining cat at necropsy but severe pulmonary lesions consistent with *D. immitis* infection were present on histopathologic examination (Table 1). Microfilariae of *D. immitis* were not detected on any day by Knott’s test of whole blood, and real time PCR for *Wolbachia* spp. on whole blood was consistently negative. Antibodies to *D. immitis* were detected by both assays in five cats on day 84, and in all six cats on study days 112, 140, 168, 196, and 224. Antigen of *D. immitis* was detected in 0, 1, and 1 cat on days 168, 196, and 224, respectively, when testing was performed according to manufacturer’s instructions, without prior heating, using the microtiter well assays. When testing was performed with membrane bound ELISA and lateral flow immunochromatographic tests, antigen was detected in 0 or 1 cat, respectively.

**Table 1:** **Results from testing feline serum samples collected 196 and 224 days after experimental infection with 100 third-stage larvae of *Dirofilaria immitis *** (**positive results in bold**)

	**Day 196**				**Day 224**			
**Cat (number of worms recovered)**	**Antigen before heating (O. D.)**	**Antigen after heating (O. D.)**	**Antibody**	**Antigen/antibody ratio**	**Antigen before heating (O. D.)**	**Antigen after heating (O. D.)**	**Antibody**	**Antigen/antibody ratio**
1 (0)*	**0.157**	**0.666**	**0.529**	1.259	0.059	**0.604**	**0.396**	1.525
2 (6)	0.070	**0.587**	**1.301**	0.451	0.099**	**1.985**	**1.605**	1.237
3 (1)	0.064	**0.289**	**0.264**	1.095	0.056	0.120**	**0.509**	0.236
4 (2)	0.055	0.081	**0.578**	0.140	0.060	0.068	**0.528**	0.129
5 (1)	0.060	**0.146**	**1.111**	0.131	0.074	**1.921**	**1.329**	1.445
6 (2)	0.050	**0.191**	**0.374**	0.511	0.057	**0.579**	**1.387**	0.417
Mean (2.0)	0.076	0.327	0.693	0.598	0.068	0.880	0.959	0.832
Number positive	1/6	5/6	6/6		1/6	5/6	6/6	

*No adult worms recovered but severe pulmonary lesions identified on histologic examination of lung sections. **Antigen was detected by visual color change but did not exceed the manufacturer’s specified cutoff (negative control + 0.05) for positive on the spectrophotometric assay used (PetChek® Heartworm PF Antigen Test, IDEXX Laboratories, Inc).

Positive results in bold.

After heat treatment of samples, the microtiter well assays identified antigen in 1, 5, and 5 of 6 cats on days 168, 196, and 224, respectively. Antigen was also detected in 4 of 6 cats after heating samples collected on day 224 using both the membrane bound ELISA and the lateral flow immunochromatographic test. Results (O.D.) on one of the microtiter plate assays (PetChek® Heartworm PF Antigen Test, IDEXX Laboratories, Inc.) before and after heating, and of antibody assay from the same samples, for days 196 and 224 are shown in Table 1. Results (O.D.) of antigen test (PetChek® Heartworm PF Antigen Test, IDEXX Laboratories, Inc.) after heating and antibody tests were significantly associated when all days were considered together (*R*^*2*^ = 0.40, *F* = 16.6, *P* < 0.001) and on samples from day 224 (*R*^*2*^ = 0.70, *F* = 16.1, *P* =0.005) but not for days 168 (*F* = 2.6, *P* =0.15) or 196 (*F* = 0.36, *P* =0.56) alone. The antigen/antibody ratio was highest in the single cat for which antigen was detected prior to heating on day 196 (Table 1), although the limited number of samples precluded meaningful statistical analysis.

## Discussion

In the present study, we showed that heat treatment of feline serum prior to antigen testing resulted in a dramatic increase in detection of *D. immitis* antigen. Although only 1/6 (16.7%) samples from cats infected with a low number of adult heartworms was antigen positive prior to heat treatment, as many as 5/6 (83.3%) became positive after heat treatment when the most sensitive microtiter well plate assays were used, presumably due to destruction of antibody and release of antigen from antibody-antigen complexes [8]. Accordingly, antigen blocking resulted in a false negative result from the majority (80%) of cats harboring both adult *D. immitis* and circulating antigen. Given the findings in the present study, it seems prudent to heat treat serum samples from cats with a high index of suspicion for heartworm infection prior to testing for antigen of *D. immitis*.

Although the American Heartworm Society feline guidelines specifically state that “antigen tests are more reliable [in cats] than generally credited,” many still have the impression that because cats generally have very low worm burdens or male-only worm infections, antigen tests are not useful in cats [4,6]. The data in the present study are exciting in that they suggest that once antigen-antibody complexes are disrupted, antigen tests may be of great value in confirming feline infection with *D. immitis*, particularly the microtiter well-based assays. Heat treatment of samples prior to testing may have clinical utility, particularly given the low level of circulating microfilariae usually present in cats and difficulties in interpreting antibody test results [2,5]. This finding may be of use not only in confirming an initial clinical suspicion of feline heartworm infection, but also for evaluating the phenomenon of self-cure in cats, which previously required evaluation of antibody levels 12 months after antigen of *D. immitis* was no longer identified [14]. The prevalence of *D. immitis* antigen in feline samples in the United States is estimated to be 0.9% [15], but the data in the present study suggest that the actual prevalence is likely higher, particularly in areas where canine heartworm infection and thus spillover into cats, is endemic or hyperendemic.

Interestingly, heat treatment of sera allowed us to detect antigen in almost every infected cat included in the present study. The basis for interference with antigen detection is not fully understood, but in other systems this process is thought to be related to antigen-antibody complex formation [16,17]. Cats infected with *D. immitis* develop a remarkable degree of inflammation which manifests as severe lung pathology characterized by villous arteritis, medial hypertrophy of the pulmonary arterioles, and pulmonary parenchymal damage, collectively referred to as heartworm-associated respiratory disease (HARD) [2,3,5]. This chronic inflammatory process likely results in hypergammaglobulinemia, which may prevent detection of *D. immitis* antigen by either specific or non-specific formation of antigen-antibody complexes *in vivo*[8,18]. Elucidation of the mechanisms responsible for inhibition of antigen detection in feline samples as well as the best approach to disrupt these complexes will require additional research.

## Conclusions

Antigen tests for *D. immitis* have long been considered of limited utility in cats. The data in the present study suggest this concept should be revisited. Heating feline serum samples prior to testing greatly facilitated detection of *D. immitis* antigen, presumably due to disruption of antigen-antibody complexes.

## Competing interests

In the past five years, SL, JT, and BB have received reimbursement, speaking fees, or research support from IDEXX Laboratories and Pfizer Animal Health (now Zoetis), manufacturers of *D. immitis* antigen tests, or from Bayer Animal Health, a company that manufactures heartworm preventives, including those for cats. In addition, JH is an employee of Bayer Animal Health. The other authors (MR, JG, JM) have no competing financial interests to disclose.

## Authors’ contributions

SL, BB, and JM conceived of the study, SL and JH participated in its design and execution and drafted the manuscript, and MR and JG carried out the antigen and antibody testing. JT led the completion of the study, particularly the experimental infections. All authors read and approved the final version of the manuscript.
